# Antibiotic Resistance Patterns in Cervical Microbes of Gilts and Sows

**DOI:** 10.3390/ani12010117

**Published:** 2022-01-04

**Authors:** Cecilia Kellerman, Pongpreecha Malaluang, Ingrid Hansson, Lena Eliasson Selling, Jane M. Morrell

**Affiliations:** 1 Clinical Sciences, Swedish University of Agricultural Sciences (SLU), P.O. Box 7054, SE-75007 Uppsala, Sweden; cecilia.kellerman@gardochdjurhalsan.se (C.K.); pongpreecha.malaluang@slu.se (P.M.); 2 Biomedical Science and Veterinary Public Health, Swedish University of Agricultural Sciences (SLU), P.O. Box 7036, SE-75007 Uppsala, Sweden; ingrid.hansson@slu.se; 3 Farm and Animal Health, Kungsängensgård, SE-75323 Uppsala, Sweden; lena.selling@gardochdjurhalsan.se

**Keywords:** antimicrobial resistance, vaginal flora of pigs, cervical swabs, antibiotics in semen extenders, antibiotic minimum inhibitory concentration

## Abstract

**Simple Summary:**

Antimicrobial resistance is occurring at an alarming rate around the world; as a result, some bacterial infections are no longer treatable with antibiotics. “Prudent use” involves only using antibiotics for therapeutic purposes. One non-therapeutic use is in semen extenders used to prepare insemination doses, which is required by law for international trade. The pig breeding industry uses large volumes of semen extenders every year. In this study, we investigate whether the antimicrobial resistance patterns of bacteria in the cervix of pigs are different in non-inseminated females compared to those that have had several litters of piglets following artificial insemination. We found that the resistance patterns in bacteria from these two groups of pigs were different, with more resistance in the pigs that had already had several litters. Some bacteria showed antibiotic resistance, and even multidrug resistance, despite no record of these antibiotics being used on the farm. These findings suggest that alternatives to antibiotics in semen extenders are required.

**Abstract:**

Extenders for boar semen contain antibiotics, which may induce antimicrobial resistance (AMR) in inseminated females. The objective was to investigate AMR of bacteria isolated from the cervix of sows and gilts in standing heat, representing females previously exposed to antibiotics in the semen extender and non-exposed females, respectively. Cervical swabs were taken from 30 multiparous sows and 30 gilts prior to their first insemination. After culturing on agar plates, bacterial isolates were identified by Matrix-Assisted Laser Desorption/Ionization Time of Flight Mass Spectrometry and antimicrobial minimum inhibitory concentrations (MIC) were determined. Differences in antibiotic resistance between sows and gilts were analyzed by Chi-squared or Fisher’s exact test. Bacteria isolated were mostly *Staphylococcus* spp., *Streptococcus* spp. and *Corynebacterium* spp. Higher MICs were observed for isolates from sows than from gilts. Most (>80%) *Corynebacterium* spp. were resistant to clindamycin; small numbers (<20%) were resistant to gentamicin, penicillin, vancomycin, ciprofloxacin and rifampicin, with no differences between gilts and sows. *Corynebacterium* from gilts were more often resistant to tetracycline than those from sows (25% vs. 4.17%; *p* = 0.04). In conclusion, bacteria from the porcine cervix showed low resistance to most antibiotics except for clindamycin, but antibacterial resistance may increase with increasing parity.

## 1. Introduction

Widespread resistance to antimicrobial substances is causing a severe problem globally when trying to treat some bacterial infections [1]. Although the size of the problem is contested by some [2], a concerted effort is being made for prudent use of antibiotics, i.e., antibiotics should only be used therapeutically and after sensitivity testing to determine which agents would be efficacious against the particular microbes involved [3].

A non-therapeutic use of antibiotics occurs in the production of semen doses for artificial insemination (AI), where they are added to semen extenders [4]. This is of particular concern in the pig breeding industry, where large numbers of sows are inseminated each year, using 80 mL extended semen per insemination dose and often two doses within one oestrus period. Since the distal urethra of the male is colonized by bacteria from skin and from the environment, these bacteria are transferred to semen during ejaculation. Other sources of contamination in semen are from the environment in the semen collection area and processing laboratory and from personnel [5]. Although strict hygiene precautions are taken during semen collection and processing, it is difficult to avoid some contamination. Bacteria can have a negative effect on sperm quality in the form of reduced sperm motility and damaged acrosomes [6,7], resulting in decreased pregnancy rates and litter size in inseminated sows. Therefore, antibiotics are added to semen extenders during processing of semen to inhibit the growth of these contaminants [8,9].

The addition of antibiotics to semen extenders is stipulated by national and international regulations, e.g., Council Directive 90/429/EEC Annex C2 of the Council of Europe [10], which states that an effective combination of antibiotics, in particular against leptospires and mycoplasmas, must be added to the semen after final dilution for export within the European Union. This combination must produce an effect at least equivalent to the following dilutions: 500 IU per ml streptomycin, 500 IU per ml penicillin, 150 mg per ml lincomycin and 300 mg per ml spectinomycin. Apart from the antibiotics already mentioned, those commonly used in boar semen extenders include gentamicin, amoxicillin, tylosin, polymixin and enrofloxacin [5].

Previously, research has focused on the boar and how bacteria commonly found in boar semen affect sperm quality, as well as the effects of antibiotics on sperm quality [8,9]. The effect of antibiotics in semen doses on the microbial flora of the inseminated sow has not been studied. It is not known whether antimicrobial resistance develops in the sow as a result of exposure to these antibiotics, or whether the resistance pattern changes with increasing parity, due to the increased number of exposures. Therefore, the objective of the present study was to investigate the pattern of antimicrobial resistance in bacteria isolated from the cervix of non-inseminated gilts and of multiparous sows that had already been inseminated on several occasions.

## 2. Materials and Methods

### 2.1. Animals and Sampling Procedure

The study population consisted of 30 sows and 30 gilts on three farms in the middle of Sweden during the autumn of 2018 (10 gilts and 10 sows per farm, Yorkshire × Landrace hybrids). Each of the farms had more than 350 sows in production and was considered tobe representative of breeding units in Sweden in terms of hygiene and husbandry. The gilts had never been inseminated prior to sampling, whereas the sows had already had 3–7 litters following AI. The animals were housed and treated according to national and international guidelines on the husbandry and care of animals. Ethical approval (number 5.8.18-15533/2018) was obtained.

The inclusion criteria were that the animals had not received any antibiotic treatment in the five weeks prior to sampling and did not have a vaginal discharge at the time of sampling. All three farms received standard semen doses extended in NutriXcell (IMV Technologies, L’Aigle, France) from the same commercial semen production center (Svenska Köttföretagen AB, Hållsta, Eskilstuna Municipality, Sweden), transported at 17–20 °C and stored at 16–18 °C for up to 5 days after semen collection. The semen extender contained antibiotics according to national and international regulations. However, the manufacturer has not reported which antibiotics are used in the extender, or their concentration.

**Figure 1:**
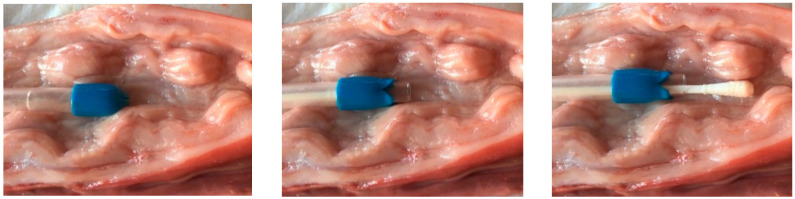
Guarded swab in the cervix of a sow’s reproductive tract (slaughterhouse material), showing the positioning of the swab for sampling and the sampling procedure itself to avoid contamination.

Using guarded swabs, samples of the reproductive tract flora were taken from the cervix at the time of standing heat, immediately prior to insemination. Before carrying out the sampling procedure on live animals, it was tested on organs obtained from a local slaughterhouse to determine how far the swab should be inserted for each category of pig to reach the caudal cervix (Figure 1).

At the farm, the vulva was wiped with a clean paper towel, the vulval lips were parted and the sterile guarded swab was gently inserted, initially in a craniodorsal direction to avoid entering the urethra and then cranially until slight resistance was felt. The cotton swab was exteriorized in two stages for sampling, rotated several times and then withdrawn into the sheath again for removal from the cervix. The swab was placed in Amies transport medium and transported at ambient temperature to the laboratory at the Swedish University of Agricultural Sciences for plating out within 6 h of sampling.

### 2.2. Microbiology: Culture, Identification of Isolates, Resistance Testing

The bacteria were cultured on bovine blood agar, lactose purple agar, mannitol salt agar (selective agar for *Staphylococcus* spp.), Colistin-Oxolinic Acid-Blood Agar (COBA; selective agar for *Streptococcus* spp.) and Man, Rogosa and Sharpe agar (MRS; selective agar for *Lactobacillus* spp.). Blood agar, blue agar and mannitol salt agar plates were incubated at 37 °C for 24 + 24 h, COBA plates were incubated at 37 °C in 5% CO_2_ for 24 + 24 h, whereas MRS-agar plates were incubated anaerobically for 5 days at 25 °C. Isolates were identified by Matrix-Assisted Laser Desorption/Ionization Time of Flight Mass Spectrometry (MALDI-TOF MS). Identification of some Gram-positive bacteria was facilitated by adding 0.1 µL formic acid to the MALDI-TOF plate before the matrix was applied.

The isolates were identified to species level by MALDI-TOF MS. The isolates were irradiated with laser UV light, which broke the molecules in the bacteria into fragments that were projected towards a detector. The time it took for the fragments to reach the detector was measured. The molecules gave rise to many fragments resulting in a characteristic mass spectrum, which was compared with stored mass spectra of known bacteria in the database (Bruker Daltonics, Billerica, MA, USA).

The identified colonies were stored in glycerolated broth (15% glycerol and 85% serum broth) at −70 °C until required for resistance testing, when they were cultured for 24 h on blood agar at 37 °C to allow pure colonies to be sub-cultured for a further 24 h. Material from 3 to 5 colonies of the pure culture was resuspended in 5 mL cation-adjusted Müller Hinton-broth (CAMHB). The broth was incubated for 130 min for *E. coli*, 4 h for *Staphylococcus* spp. and 3.5 h for *Streptococcus* spp. in 50 µL aliquots in a microtiter plate. Bacterial concentration in the solution was checked by transferring 10 µL to 10 mL 0.9% NaCl; 100 µL of this solution was plated out on blood agar and incubated at 36 °C for 18 h, after which time 20–80 colonies were expected to be seen. The minimum inhibitory concentration (MIC) of each antibiotic was determined to be the concentration that prevented visible growth of bacteria on the plate.

Isolates of *Corynebacterium* spp. were tested for susceptibility to selected antimicrobial substances by minimum inhibitory concentration (MIC) according to the standard procedures of the Clinical Laboratory Standards Institute [11] assessed with panel analysis systems: for *E. coli*, VetMIC™ GN-mo, for *Staphylococcus* spp. and *Streptococcus* spp. VetMIC™ CLIN Staph/Strept and for *Lactobacillus* spp. VetMIC™ Lact-1 and VetMIC™ Lact-2 (National Veterinary institute [SVA], Uppsala, Sweden). For quality control, *E. coli* ATCC 25922 was used for Gram-negative bacteria, i.e., *E. coli,* and *Staphylococcus aureus* ATCC 29213 for Gram-positive bacteria, i.e., *Staphylococcus* spp., *Streptococcus* spp. and *Lactobacillus* spp.

The epidemiological breakoff point (ECOFF) established by the European Committee for testing of antibiotic resistance (EUCAST) [12] was used for determination of resistance. The ECOFF values classify isolates with acquired reduced susceptibility as ‘non-wild type’. In this paper, non-wild type isolates are called ‘resistant’, in agreement with the Swedish Veterinary Antibiotic Resistance Monitoring report (Swedres-SVARM 2020 [13]). This classification is relevant for monitoring purposes, but it should be understood that resistance defined in this manner does not always refer to clinical resistance.

### 2.3. Statistical Analysis

The proportions of susceptible and resistant *Corynebacterium* spp. isolates to each antibiotic were compared between gilts and sows and between farms. The comparison of relative frequencies was performed with a Chi-squared test. Fisher’s exact test was used for low cell counts. Differences were considered significant if *p* ≤ 0.05.

## 3. Results

### 3.1. Isolates

Bacteria were isolated from all animals. In total, 280 isolates were identified from 48 strains, belonging to three phyla: *Firmicutes* (174 isolates) *Actinobacteria* (82 isolates) and *Proteoacteria* (24 isolates). Of these, 72.9% belonged to three genera: *Staphylococcus*, *Streptococcus* and *Corynebacterium* (Table 1). *Staphylococcus* spp. (Table 1) were isolated from 55 of the 60 samples, *Streptococcus* spp. from 49 of the 60 samples, and *Corynebacterium* spp. from 40 of the 60 samples. In addition, *Bacillus* spp. were isolated from 12 samples, *Micrococcus* spp. from 6 samples, *Enterococcus* spp. from 3 samples and *Pasteurella* spp. from 2 samples. There were no differences between farms.

### 3.2. Resistance Determination

Resistance determination was carried out on *E. coli*, *S. chromogenes*, *S. lentus*, *S. rostris*, *S. suis*, *S. hyovaginalis* and *S. thoraltensis*. However, it was not possible to interpret the results from the latter two species because of insufficient growth. Only one colony of *S. hyovaginalis* was seen after 11 h incubation and dilution according to the instructions. Similarly, for *S. thoraltensis*, only seven colonies were seen after incubation (Table 2).

The distribution of MICS (mg/L) and resistance of specific bacteria are provided in Supplementary Tables S1–S7. The ECOFF-values are marked with a vertical line for each antibiotic (or closely related antibiotic if a value is not included in EUCAST table [11]).

All isolates showed a varying degree of sensitivity to different antibiotics, regardless of the reproductive status of the animals from which they originated. There were some differences in the resistance patterns of bacteria between gilts and sows. Thus, higher concentrations of tetracycline and penicillin were needed to inhibit growth of *S. rostri* in gilts than in sows (Table S4), whereas higher concentrations of penicillin were required to inhibit growth of *S. chromogenes* and *S. sciuri* in sows than in gilts (Supplementary Tables S2 and S5, respectively).

Most (>80%) *Corynebacterium* spp. (Supplementary Table S7) were resistant to clindamycin, with no difference between gilts (85.7%) and sows (95.8%). Small numbers (<20.0%) were resistant to gentamicin (3.6 and 4.2%, for gilts and sows, respectively), penicillin (10.7%; 12.5%), vancomycin (3.6%; 4.2%), ciprofloxacin (3.6%; 4.2%) and rifampicin (0%; 4.2%), again with no significant differences between gilts and sows. However, *Corynebacterium* isolated from gilts were more often resistant to tetracycline compared with *Corynebacterium* from sows (25.0%; 4.2%; *p* = 0.04). None of the *Corynebacterium* showed any resistance to linezolid. Multidrug resistance was detected in both gilts and sows, with four and two isolates of *Corynebacterium* spp., respectively, and five isolates of *Staphylococcus* spp. each from gilts and sows being resistant to three or more antibiotics.

## 4. Discussion

The purpose of this study was to examine the occurrence of antimicrobial resistance in the bacterial flora of the porcine cervix. Some differences were observed between gilts and sows, i.e., between females that had not previously been inseminated and those that had already had several litters of piglets following AI.

The bacteria isolated in this study were mostly *Staphylococcus* spp., *Streptococcus* spp. and *Corynebacterium* spp., in agreement with a previous study in Queensland [14]. However, in contrast, fewer *E. coli* were isolated in our study than in the Queensland study, although this could be due to the timing of sampling. In the study by Bara et al. [14], the sampling was performed in conjunction with parturition and mating, when the reproductive tract is exposed to environmental bacteria, whereas in the present study, it was performed immediately prior to AI, approximately five weeks after parturition in the case of the sows. By this time, bacteria contaminating the uterus, cervix and vagina during parturition should have been removed. The hygiene conditions in the barn also play an important role. In the present study, the animals were group housed, bedded on straw, in clean conditions. In contrast, in the study in Queensland, the sows were housed in individual stalls in contact with their own excrement. Another explanation could be that *E. coli* would be more likely to be found in the caudal vagina, with the chances of isolation decreasing if the samples are taken from a more cranial position. The isolation of so few *E. coli* from our samples (7 out of 60 samples) also indicates that wiping the vulva only with dry paper towel before taking the sample is sufficient to avoid contamination from the vulva, provided that a guarded swab is used. However, additional cleaning may be required if the sanitary conditions are poor.

Bacteria from the phylum *Acinetobacter* were more common in sows than in gilts (32.2% and 22.2%, respectively). The main species isolated was *Actinomyces hyovaginalis*, which occurred to approximately the same extent on all farms. In other respects, there was little difference in the types of bacteria isolated from gilts and sows, apart from some sows having a more varied flora than gilts.

Since *Lactobacillus* spp. appear in the flora of several species, such as cows [15], horses [16] and human beings [17], it was expected that they would be isolated in our study, but none were found. In one of the gilts, one isolate was identified as *L. delbrueckii* but with a MALDI-TOF score of 1.79, which is not sufficient for a definite identification. This particular isolate grew very slowly, possibly indicating that the conditions for culturing these bacteria may not have been optimal in spite of the selective medium used. If this is the case, then *Lactobacillus* spp. may also be found in the cervix of pigs. It is also possible that more *Lactobacillus* spp. would have been isolated if the sampling had been performed in the caudal vagina instead of in the cervix.

Tetracycline resistance of *Corynebacterium* was higher in gilts than sows although there were no obvious explanations for this finding (such as administration of tetracycline to the gilts). We speculate that it may be due to contact with this antibiotic during the piglets’ early life, followed by waning of resistance with time. Tetracycline is not commonly added to semen extenders.

There was, however, some evidence that resistance could develop in response to the antibiotics present in semen extenders, because higher concentrations of certain antibiotics were needed to retard bacterial growth in isolates from sows than from gilts, e.g., for *S. sciuri* and *S. chromogenes,* where the sows had been inseminated on several occasions. However, the opposite was true, e.g., for *S. lentus* where a higher concentration of antibiotics was needed to inhibit the growth of isolates from gilts than from sows.

In all *Staphylococcus* spp. there was a tendency for sensitivity to penicillin to be decreased in the samples from sows compared with those from gilts. However, the number of animals was too small to detect significant differences. According to Gadea [18], commonly used combinations of antibiotics in extenders for boar semen include penicillin and streptomycin (1 g/L), aminoglycosides such as gentamicin, neomycin and kanamycin at concentrations of approximately 200 mg/L, or third generation cephalosporins (ceftiofur, apramycin, etc.).

It is not known why the resistance to clindamycin should be so high among animals from all farms. There was no record of this antibiotic being used on any of the farms, and it is not one of the antibiotics commonly added to semen extenders. It is possible that treatment of human patients with this antibiotic, e.g., for throat infections, resulted in resistance appearing in environmental bacteria, possibly via disposal of sewage effluent, and hence appearing in the cervical flora of pigs. This speculated route of resistance development enhances the need for prudent use of antibiotics in both medical and veterinary fields. However, it should be noted that bacteria evolve to enable them to survive in a hostile environment [19]; the genes that facilitate survival to such environments may not be specifically for resistance to antibiotics, but such resistance is conferred serendipitously (for the bacteria).

After insemination, some fluid is expelled from the vagina via backflow [20], thereby exposing the bacteria in the animals’ environment to antibiotics, with the potential for selection of resistance genes. An investigation of this effect on resistance patterns in environmental bacteria would be interesting but might be difficult to conduct.

Our results indicate that antibiotic resistance is found among vaginal microbes in both gilts and sows and that the pattern of resistance differs between isolates from gilts and sows, depending on bacterial species. It was interesting, and possibly alarming, that antimicrobial resistance, and even multidrug resistance, was detected in these isolates, despite no administration of antibiotics to the animals on these farms, as reported in the farm records. It is interesting to speculate on the origin of this changing resistance pattern, since antibiotic usage in Sweden is generally low. However, the inclusion of antibiotics in the semen extender used to prepare the semen doses is a likely candidate as the source of antimicrobial resistance in the vaginal flora. The exposure of environmental bacteria to antibiotics via backflow from the inseminated sows [20] could act as a source of resistant bacteria in the environment, which then spread resistance genes to the flora in the cervix. It would be interesting to investigate if the occurrence of AMR in vaginal flora was associated with increasing parity following AI, but our sample size was not large enough to conduct such an analysis.

The fact remains that development of resistance to antibiotics is a serious threat to both human and animal health and that usage of antibiotics for non-therapeutic purposes is at best controversial and at worst imprudent. It is known that using antibiotics allows resistant bacteria to be selected and that the genes for resistance can spread to other bacteria, including pathogens. This subject is of high priority, and the EU has worked strenuously for the last 20 years to slow the development of resistance. In 2017, a new action plan against antimicrobial resistance within the EU was developed, including a ban on the use of antimicrobials for non-therapeutic purposes from 2022. This policy has already been in place in Sweden since 1986.

Breeding pigs by AI facilitates batch production with low risk of infection and consequently decreased need for antibiotics. It would be unfortunate for pig producers if the inclusion of antibiotics in semen extenders was forbidden before alternatives could be found. With the knowledge that currently exists, it is surprising that the alternatives to addition of antibiotics that have already been identified as a means of reducing bacteria in semen doses [21,22] have not received more attention and resources so that they can be scaled-up for widespread use.

## 5. Conclusions

The bacteria isolated in this study from all animals were mostly *Staphylococcus* spp., *Streptococcus* spp. and *Corynebacterium* spp. and were evenly distributed among individuals and farms. *Acinetobacter* spp. were more common in sows than in gilts. A higher concentration of antibiotics was required to slow bacterial growth in isolates from sows than from gilts, which might suggest that resistance in the cervical flora could develop in response to the antibiotics in semen extenders. Additional studies are required for verification. *Corynebacterium* from both gilts and sows showed low resistance to most of the antibiotics tested, with the exception of clindamycin. More isolates from gilts were resistant to tetracycline compared with isolates from sows.

## Figures and Tables

**Table 1:** Distribution of bacteria isolated from the cervix of 30 healthy sows and 30 healthy gilts.

Genus	Species	Sows	Gilts	Total
*Staphylococcus*		42	43	85
	*S. lentus*	10	10	20
	*S. sciuri*	9	8	17
	*S. chromogenes*	4	12	16
	*S. rostri*	7	3	10
	*S. epidermidis*	3	2	5
	*S. equorum*	1	3	4
	*S. gallinarum*	3	1	4
	*S. xylosus*	2	1	3
	*S. hominis*	1	1	2
	*S. simulans*	1	1	2
	*S. haemolyticus*	0	1	1
	*S. hyicus*	1	0	1
*Streptococcus*		34	35	69
	*S. thoraltensis*	12	16	28
	*S. suis*	11	13	24
	*S. hyovaginalis*	7	3	10
	*S. orisratti*	2	0	2
	*S. pluranimalium*	1	1	2
	*S. canis*	1	0	1
	*S. dysgalactiae*	0	1	1
	*S. hyointestinalis*	0	1	1
*Corynebacterium*		24	26	50
	*C. xerosis*	12	15	27
	*C. freneyi*	9	6	15
	*C. glucuronolyticum*	1	2	3
	*C. auricosum*	1	0	1
	*C. casei*	0	1	1
	*C. confusum*	0	1	1
	*C. glutamicum*	1	0	1
	*C. stationis*	0	1	1
*Actinomyces*	*A. hyovaginalis*	4	15	19
*Actinobacillus*	*A. rossii*	6	7	13
*Bacillus*		7	6	13
	*B. licheniformis*	6	3	9
	*B. pumilus*	1	2	3
	*B. flexus*	0	1	1
*Escherichia*	*E. coli*	3	4	7
*Micrococcus*		0	6	6
	*M. luteus*	0	5	5
	*M. tereus*	0	1	1
*Rothia*	*R. nasimurium*	1	5	6
*Enterococcus*		2	1	3
	*E. aquimarinus*	1	0	1
	*E. avium*	1	0	1
	*E. cecorum*	0	1	1
*Pasteurella*		2	0	2
	*P. mairii*	1	0	1
	*P. aerogenes*	1	0	1
*Aerococcus*	*A. viridans*	2	0	2
*Clostridium*	*C. perfringens*	1	0	1
*Globicatella*	*G. sanguinis*	1	0	1
*Pantoea*	*P. agglomerans*	1	0	1
*Proteus*	*P. mirabilis*	1	0	1
*Trueperella*	*T. pyogenes*	0	1	1

**Table 2:** Occurrence of resistance in bacterial isolates from the cervix of sows and gilts (*E.coli*, *Staphylococcus* spp. and *Streptococcus suis*).

Bacterium	Sows		Gilts	
Species (No. Isolates)	No. Isolates	Resistance	No. Isolates	Resistance
*Corynebacteríum* spp. (52)	24	1 (Cl + Pc + Va + Ri) *	28	1 (Gm + Tc + Cl + Pc + Va + Ci) *
		1 (Gm + Cl + Pc) *		1 (Tc + Cl + Pc) *
		1 (Cl + Pc)		2 (Tc + Cl + Ri) *
		1 (Tc + Cl)		2 (Cl + Ri)
		1 (Tc + Ci)		2 (Tc + Cl)
		18 (Cl)		1 (Cl + Pc)
				15 (Cl)
				1 (Pc)
				1 (Tc)
*E. coli* (7)	3	1 (Cs)	4	0
*Staph. chromogenes* (16)	4	3 (Pc)	12	2 (Em + Gm)
				1 (Pc + Cl)
				1 (Pc)
				1 (Cl)
*Staph. lentus* (20)	10	1 (Pc + Ox + Cl) *	10	1 (Fu + Cl + Gm) *
		1 (Fu + Em + Cl) *		1 (Fu + Em + Cl) *
		2 (Fu + Cl)		2 (Fu + Cl)
		2 (Ox + Cl)		1 (Cl + Tc)
		3 (Cl)		4 (Cl)
		1 (Gm)		1 (Tc)
*Staph. rostri* (10)	7	1 (Pc + Tc)	3	2 (Pc + Tc)
		1 (Pc)		
		2 (Tc)		
*Staph. sciuri (17)*	9	1 (Ox + Fu + Cl) *	8	1 (Ox + Fu + Cl) *
		1 (Ox + Fu)		3 (Ox + Fu)
		7 (Fu)		3 (Fu)
*Strept. suis* (14)	7	1 (Fox + Em + Ni + Tc) *	6	1 (Fox + Em + Cl + Ni + Tc) *
		1 (Fox + Em + Ni) *		1 (Fox + Ef + Em) *
		1 (Fox + Ni + Tc) *		2 (Fox + Tc)
		2 (Fox + Tc)		1(Fox + Em)
		2 (Fox)		

* Multidrug resistance, i.e., resistant to three or more antibiotic classes. Ci = ciprofloxacin; Cl = clindamycin; Cs = colistin; Ef = enrofloxacin; Em = erythromycin; Fox = cefoxitin; Fu = fucidic acid; Gm = gentamycin; Ni = nitrofurantoin; Ox = oxacillin; Pc = penicillin; Ri = rifampicin; Tc = tetracycline; Va = vancomycin.

## Data Availability

All data are provided in the article.

## Supplementary Materials

The following supporting information can be downloaded at: https://www.mdpi.com/article/10.3390/ani12010117/s1, Table S1: Distribution of MICs (mg/L) and resistance of *Escherichia coli* isolated from the cervix of gilts and sows. Results shown are the proportion of isolates at the MIC values for the different antibiotics (%); Table S2: Distribution of MICs (mg/L) and resistance of *Staphylococcus chromogenes* isolated from the cervix of gilts and sows. The results shown are proportions of isolates at the MIC values for the different antibiotics (%); Table S3: Distribution of MICS (mg/L) and resistance of *Staphylococcus lentus* isolated from the cervix of gilts and sows. The results are shown as proportions of isolates at the MIC values for the different antibiotics (%); Table S4: Distribution of MICs (mg/L) for *Staphylococcus rostri* isolated from the cervix of gilts and sows. The results are shown as proportions of isolates at the MIC values for the different antibiotics (%); Table S5: Distribution of MICs (mg/L) for *Staphylococcus sciuri* isolated from the cervix of gilts and sows. The results are shown as proportions of isolates at the MIC values for the different antibiotics (%); Table S6: Distribution of MICs (mg/L) for *Streptococcus suis* isolates from the cervix of gilts and sows. The results are shown as proportions of isolates at the MIC values for the different antibiotics (%); Table S7: Distribution of MICs (mg/L) and resistance of *Corynebacterium* spp. isolated from the cervix of gilts and sows. The results are shown as the proportion of isolates at the MIC values for the different antibiotics (%).
