# Supplementary material for: Antibiotic Resistance Patterns in Cervical Microbes of Gilts and Sows
Source: Animals (Basel). 2022 Jan 4;12(1):117. doi: 10.3390/ani12010117 (PMC8749660; doi:10.3390/ani12010117)
Supplement: Supplementary file 1 [file animals-12-00117-s001.zip › animals-1537300-supplementary.pdf]

Supplementary Table S1: Distribution of MICs (mg/L) and resistance of *Escherichia coli* isolated from the cervix of gilts and sows. Results shown are the proportion of isolates at the MIC values for the different antibiotics (%).

Source Animal	Antibiotic	Res	<0.12	0.25	0.5	1	2	4	8	16	32	≥64
Sows (n = 3)	Ampicillin	0					66.7	33.3				
Gilts (n = 4)		0					75.0	25.0				
Sows (n = 3)	Amoxicillin/	0						100.0				
Gilts (n = 4)	(Clavulan acid)	0						100.0				
Sows (n = 3)	Cefotaxime	0		100.0								
Gilts (n = 4)		0		100.0								
Sows (n = 3)	Colistin	33.3			66.7			33.3				
Gilts (n = 4)		0			75.0		25.0					
Sows (n = 3)	Enrofloxacin	0	100									
Gilts (n = 4)		0	100									
Sows (n = 3)	Gentamycin	0					100.0					
Gilts (n = 4)		0					100.0					
Sows (n = 3)	Neomycin	0						100.0				
Gilts (n = 4)		0						100.0				
Sows (n = 3)	Nitrofurantoin	0							33.3	66.7		
Gilts (n = 4)		0							25.0	75.0		
Sows (n = 3)	Streptomycin	0							66.7	33.3		
Gilts (n = 4)		0							50.0	50.0		
Sows (n = 3)	Tetracycline	0					66.7	33.3				
Gilts (n = 4)		0					100.0					
Sows (n = 3)	Trimetoprim/	0			100.0							
Gilts (n = 4)	(sulfametoxazol)	0			100.0							

Notes: White fields denote the range of dilutions tested for each antibiotic and grey field dilutions of antibiotic not tested; vertical bold lines indicate cut-off values used to define resistance. The MICs equal to or lower than the lowest concentration tested are given as the lowest tested concentration.

Supplementary Table S2: Distribution of MICs (mg/L) and resistance of *Staphylococcus chromogenes* isolated from the cervix of gilts and sows. The results shown are proportions of isolates at the MIC values for the different antibiotics (%).

Source Animal	Antibiotic	Res	<0.03	0.06	0.12	0.25	0.5	1	2	4	8	16	32	64
Sows (n = 4)	Cephalotin	0						100.0						
Gilts (n = 12)		0						100.0						
Sows (n = 4)	Cefoxitin	0					100							
Gilts (n = 12)		0					83.3	16.7						
Sows (n = 4)	Clindamycin	0					100.0							
Gilts (n = 12)		8.3					91.7	8.3						
Sows (n = 4)	Enrofloxacin	0				100.0								
Gilts (n = 12)		0				100.0								
Sows (n = 4)	Erythromycin	0					100.0							
Gilts (n = 12)		8.3					91.7		8.3					
Sows (n = 4)	Fucidic acid	0					100.0							
Gilts (n = 12)		0					100.0							
Sows (n = 4)	Gentamycin	0						100.0						
Gilts (n = 12)		8.3						91.7	8.3					
Sows (n = 4)	Oxacillin	0					100.0							
Gilts (n = 12)		0				66.7	33.3							
Sows (n = 4)	Penicillin	75.0	25.0			50.0		25.0						
Gilts (n = 12)		8.3	83.3					8.3						
Sows (n = 4)	Nitrofurantoin	0										100.0		
Gilts (n = 12)		0										100.0		
Sows (n = 4)	Tetracycline	0				100.0								
Gilts (n = 12)		0				91.7	8.3							
Sows (n = 4)	Trimetoprim/	0				75.0	25.0							
Gilts (n = 12)	(sulfametoxazol)	0				75.0	25.0							

Notes: White fields denote the range of dilutions tested for each antibiotic and grey field dilutions of antibiotic not tested; vertical bold lines indicate cut-off values used to define resistance. The MICs equal to or lower than the lowest concentration tested are given as the lowest tested concentration. ECOFF-values are provided for coagulase-negative *Staphylococcus* spp.

Supplementary Table S3: Distribution of MICs (mg/L) and resistance of *Staphylococcus lentus* isolated from the cervix of gilts and sows. The results are shown as proportions of isolates at the MIC values for the different antibiotics (%).

**Source Animal**	**Antibiotic**	**Res**	**<0.03**	**0.06**	**0.12**	**0.25**	**0.5**	**1**	**2**	**4**	**8**	**16**	**32**	**64**
Sows (n = 10)	Cephalotin	0						100.0						
Gilts (n = 10)		0						100.0						
Sows (n = 10)	Cefoxitin	0						30.0	60.0	10.0				
Gilts (n = 10)		0						50.0	50.0					
Sows (n = 10)	Clindamycin	90.0				10.0		50.0	40.0					
Gilts (n = 10)		80.0				10.0	10.0	70.0	10.0					
Sows (n = 10)	Enrofloxacin	0				90.0	10.0							
Gilts (n = 10)		0				10.0								
Sows (n = 10)	Erythromycin	10.0					90.0		10					
Gilts (n = 10)		10.0					80.0	10.0	10					
Sows (n = 10)	Fucidic acid	90.0					10.0	60.0	30.0					
Gilts (n = 10)		100.0						60.0	40.0					
Sows (n = 10)	Gentamycin	10.0						90.0		10.0				
Gilts (n = 10)		10.0						90.0		10.0				
Sows (n = 10)	Oxacillin	70.0					30.0	70.0						
Gilts (n = 10)		90.0					10.0	90.0						
Sows (n = 10)	Penicillin	10.0	10.0	60.0	20.0			10.0						
Gilts (n = 10)		0		80.0	20.0									
Sows (n = 10)	Nitrofurantoin	0										100.0		
Gilts (n = 10)		0										100.0		
Sows (n = 10)	Tetracycline	0				100.0								
Gilts (n = 10)		20.0				70.0	10.0			20.0				
Sows (n = 10)	Trimetoprim/	20.0				60.0	20.0	20.0						
Gilts (n = 10)	(sulfametoxazol)	0				100.0								

Notes: White fields denote the range of dilutions tested for each antibiotic and grey field dilutions of antibiotic not tested; vertical bold lines indicate cut-off values used to define resistance. The MICs equal to or lower than the lowest concentration tested are given as the lowest tested concentration. ECOFF-values are provided for coagulase-negative *Staphylococcus* spp.

Supplementary Table S4: Distribution of MICs (mg/L) for *Staphylococcus rostri* isolated from the cervix of gilts and sows. The results are shown as proportions of isolates at the MIC values for the different antibiotics (%).

Source Animal	Antibiotic	Res	<0.03	0.06	0.12	0.25	0.5	1	2	4	8	16	32	64
Sows (n = 7)	Cephalotin	0						100.0						
Gilts (n = 3)		0						100.0						
Sows (n = 7)	Cefoxitin	0					57.1	42.9						
Gilts (n = 3)		0				33.4	33.3	33.3						
Sows (n = 7)	Clindamycin	0					100.0							
Gilts (n = 3)		0					100.0							
Sows (n = 7)	Enrofloxacin	0				71.4								
Gilts (n = 3)		0				100.0								
Sows (n = 7)	Erythromycin	0					100.0							
Gilts (n = 3)		0					100.0							
Sows (n = 7)	Fucidic acid	0					100.0							
Gilts (n = 3)		0					100.0							
Sows (n = 7)	Gentamycin	0						100.0						
Gilts (n = 3)		0						100.0						
Sows (n = 7)	Oxacillin	0				100.0								
Gilts (n = 3)		0				100.0								
Sows (n = 7)	Penicillin	28.6	57.1	14.3		14.3		14.3						
Gilts (n = 3)		66.7	33.3			66.7								
Sows (n = 7)	Nitrofurantoin	0											100.0	
Gilts (n = 3)		0											100.0	
Sows (n = 7)	Tetracycline	42.8				28.6	28.6		28.6	14.2				
Gilts (n = 3)		66.7				33.3				66.7				
Sows (n = 7)	Trimetoprim/	0				28.6	14.3		14.3	42.8				
Gilts (n = 3)	(sulfametoxazol)	0				33.3				66.7				

Notes: White fields denote the range of dilutions tested for each antibiotic and grey field dilutions of antibiotic not tested; vertical bold lines indicate cut-off values used to define resistance. The MICs equal to or lower than the lowest concentration tested are given as the lowest tested concentration. ECOFF-values are provided for coagulase-negative *Staphylococcus* spp.

Supplementary Table S5: Distribution of MICs (mg/L) for *Staphylococcus sciuri* isolated from the cervix of gilts and sows. The results are shown as proportions of isolates at the MIC values for the different antibiotics (%).

Animal Source	Antibiotic	Res	<0.03	0.06	0.12	0.25	0.5	1	2	4	8	16	32	64
Sows (n = 9)	Cefalotin	0						100.0						
Gilts (n = 8)		0						100.0						
Sows (n = 9)	Cefoxitin	0							66.7	33.3				
Gilts (n = 8)		0						12.5	50.0	37.5				
Sows (n = 9)	Clindamycin	11.1				88.9		11.1						
Gilts (n = 8)		12.5				87.5		12.5						
Sows (n = 9)	Enrofloxacin	0				44.4	55.6							
Gilts (n = 8)		0				12.5	87.5							
Sows (n = 9)	Erythromycin	0					100.0							
Gilts (n = 8)		0					100.0							
Sows (n = 9)	Fucidic acid	100.0							100.0					
Gilts (n = 8)		87.5					12.5		87.5					
Sows (n = 9)	Gentamycin	0						100.0						
Gilts (n = 8)		0						100.0						
Sows (n = 9)	Oxacillin	100.0						100.0						
Gilts (n = 8)		100.0						100.0						
Sows (n = 9)	Penicillin	0		33.3	66.7									
Gilts (n = 8)		0		75.0	25.0									
Sows (n = 9)	Nitrofurantoin	0										100.0		
Gilts (n = 8)		0										100.0		
Sows (n = 9)	Tetracycline	0				88.9	11.1							
Gilts (n = 8)		0				100.0								
Sows (n = 9)	Trimetoprim/	0				88.9	11.1							
Gilts (n = 8)	(sulfametoxazol)	0				100.0								

Notes: White fields denote the range of dilutions tested for each antibiotic and grey field dilutions of antibiotic not tested; vertical bold lines indicate cut-off values used to define resistance. The MICs equal to or lower than the lowest concentration tested are given as the lowest tested concentration. ECOFF-values are provided for coagulase-negative *Staphylococcus* spp.

Supplementary Table S6: Distribution of MICs (mg/L) for *Streptococcus suis* isolates from the cervix of gilts and sows. The results are shown as proportions of isolates at the MIC values for the different antibiotics (%).

		Res	<0.03	0.06	0.12	0.25	0.5	1	2	4	8	16	32	64
Sows (n = 7)	Cephalotin	0						100.0						
Gilts (n = 6)		0						100.0						
Sows (n = 7)	Cefoxitin	0						28.6	14.3	57.1				
Gilts (n = 6)		0					16.7		50.0	33.3				
Sows (n = 7)	Clindamycin	0					100.0							
Gilts (n = 6)		16.7					83.3		16.7					
Sows (n = 7)	Enrofloxacin	0				28.6	71.4							
Gilts (n = 6)		0				33.3	50.0	16.7						
Sows (n = 7)	Erythromycin	28.6				71.4		14.3	14.3					
Gilts (n = 6)		50.0				50.0		50.0						
Sows (n = 7)	Fucidic acid	NA							100.0					
Gilts (n = 6)		NA							100.0					
Sows (n = 7)	Gentamycin	NA						85.7	14.3					
Gilts (n = 6)		NA						83.8	16.7					
Sows (n = 7)	Oxacillin	0				100.0								
Gilts (n = 6)		0				100.0								
Sows (n = 7)	Penicillin	0	14.2	42.9	42.9									
Gilts (n = 6)		0	16.7	33.3	50.0									
Sows (n = 7)	Nitrofurantoin	0										57.1	42.9	
Gilts (n = 6)		0										83.3	16.7	
Sows (n = 7)	Tetracycline	57.1					28.6	14,3		57.1				
Gilts (n = 6)		50.0				16.6	16.7	16.7		50.0				
Sows (n = 7)	Trimetoprim/	0				42.9	57.1							
Gilts (n = 6)	(sulfametoxazol)	16.7				33.3	50.0	16,7						

Notes: White fields denote the range of dilutions tested for each antibiotic and grey field dilutions of antibiotic not tested; vertical bold lines indicate cut-off values used to define resistance. The MICs equal to or lower than the lowest concentration tested are given as the lowest tested concentration. ECOFF-values for alpha-haemolytic *Streptococcus* spp. are shown unless otherwise specified.

Supplementary Table S7: Distribution of MICs (mg/L) and resistance of *Corynebacterium* spp. isolated from the cervix of gilts and sows. The results are shown as the proportion of isolates at the MIC values for the different antibiotics (%).

		**Res**	**0.12**	**0.25**	**0,5**	**1**	**2**	**4**	**8**	**16**	**32**	**64**	**128**	**256**
Sows (n = 24)	Gentamycin	4.2				95.8	4.2							
Gilts (N = 28)		3.6				96.4		3.6						
Sows (n = 24)	Kanamycin	NA						100.0						
Gilts (N = 28)		NA						96.4					3.6	
Sows (n = 24)	Streptomycin	NA				45.8	37.4	4.2	4.2	4.2		4.2		
Gilts (N = 28)		NA				35.7	57.1				3.6		3.6	
Sows (n = 24)	Neomycin	NA				100.0								
Gilts (N = 28)		NA				96.4		3.6						
Sows (n = 24)	Tetracycline	4.2			8.3	66.7	20.8			4.2				
Gilts (N = 28)		25.0		3.6	10.7	35.7	25.0	3.6	3.6	10.7	7.1			
Sows (n = 24)	Erythromycin	NA	91.6		4.2			4.2						
Gilts (N = 28)		NA	71.5			3.6	7.1	7.1	10.7					
Sows (n = 24)	Clindamycin	95.8	4.2			45.8	37.5	12.5						
Gilts (N = 28)		85.7	3.6		10.7	46.4	17.9	7.1	14.3					
Sows (n = 24)	Chloramphenicol	NA					12.5	70.8	4.2	8.3	4.2			
Gilts (N = 28)		NA					35.7	57.1	3.6		3.6			
Sows (n = 24)	Ampicillin	NA	87.4	0	8.4			4.2						
Gilts (N = 28)		NA	89.3	10.7										
Sows (n = 24)	Penicillin	12.6	87.4	4.2	4.2		4.2							
Gilts (N = 28)		10.7	89.3	10.7										
Sows (n = 24)	Vancomycin	4.2			95.8			4.2						
Gilts (N = 28)		3.6			96.4								3.6	
Sows (n = 24)	Quinupristin	NA		4.2	45.8	33.3	12.5	4.2						
Gilts (N = 28)	/Dalfopristin	NA		7.2	57.1	14.3	7.1	7.1	7.2					
Sows (n = 24)	Linezolid	0			50.0	50.0								
Gilts (N = 28)		0			39.3	57.1	3.6							
Sows (n = 24)	Trimethoprim	NA				8.3	25.0	25.0	41.7					
Gilts (N = 28)		NA				10.7	39.3	17.9	17.9	7.1	3.6	3.6		
Sows (n = 24)	Ciprofloxacin	4.2			50.0	45.8	4.2							
Gilts (N = 28)		3.6			39.3	57.1	3.6							
Sows (n = 24)	Rifampicin	4.2		95.8					4.2					
Gilts (N = 28)		0		100.0										

White fields denote the range of dilutions tested for each antibiotic and grey field dilutions of antibiotic not tested, and vertical bold lines indicate cut-off values used to define resistance. MICs equal to or lower than the lowest concentration tested are given as the lowest tested concentration. NA, not applicable since no cut off value is given.
